# Transcriptome analysis of *Petunia axillaris* flowers reveals genes involved in morphological differentiation and metabolite transport

**DOI:** 10.1371/journal.pone.0198936

**Published:** 2018-06-14

**Authors:** Ikuko Amano, Sakihito Kitajima, Hideyuki Suzuki, Takao Koeduka, Nobukazu Shitan

**Affiliations:** 1 Laboratory of Medicinal Cell Biology, Kobe Pharmaceutical University, Motoyamakita-machi, Higashinada-ku, Kobe, Japan; 2 Department of Applied Biology, Kyoto Institute of Technology, Matsugasaki Sakyo-ku, Kyoto, Japan; 3 The Center for Advanced Insect Research Promotion, Kyoto Institute of Technology, Matsugasaki Sakyo-ku, Kyoto, Japan; 4 Department of Research and Development, Kazusa DNA Research Institute, Chiba, Japan; 5 Graduate School of Sciences and Technology for Innovation (Agriculture), Department of Biological Chemistry, Yamaguchi University, Yamaguchi, Japan; University of Naples Federico II, ITALY

## Abstract

The biosynthesis of plant secondary metabolites is associated with morphological and metabolic differentiation. As a consequence, gene expression profiles can change drastically, and primary and secondary metabolites, including intermediate and end-products, move dynamically within and between cells. However, little is known about the molecular mechanisms underlying differentiation and transport mechanisms. In this study, we performed a transcriptome analysis of *Petunia axillaris* subsp. *parodii*, which produces various volatiles in its corolla limbs and emits metabolites to attract pollinators. RNA-sequencing from leaves, buds, and limbs identified 53,243 unigenes. Analysis of differentially expressed genes, combined with gene ontology and Kyoto Encyclopedia of Genes and Genomes pathway analyses, showed that many biological processes were highly enriched in limbs. These included catabolic processes and signaling pathways of hormones, such as gibberellins, and metabolic pathways, including phenylpropanoids and fatty acids. Moreover, we identified five transporter genes that showed high expression in limbs, and we performed spatiotemporal expression analyses and homology searches to infer their putative functions. Our systematic analysis provides comprehensive transcriptomic information regarding morphological differentiation and metabolite transport in the *Petunia* flower and lays the foundation for establishing the specific mechanisms that control secondary metabolite biosynthesis in plants.

## Introduction

Plants produce various secondary metabolites to adapt to their environments. Most of these metabolites are produced only in specific organs, tissues, and cells, where biosynthetic enzymes are expressed. Therefore, the proper timing and regulation of these enzymes is associated with morphological and metabolic differentiation of plant organs [1].

Flowers are specialized organs that are morphologically and metabolically differentiated. Most flowers have sepals, petals, and reproductive organs (pistils and stamens). In the petals, pigments are produced and accumulate, whereas volatiles are produced and emitted. In some plants, morphology and secondary metabolites play important roles in reproductive success. For example, in the South American genus *Petunia*, floral phenotype and pollinators have coevolved [2]. *Petunia integrifolia* flowers have short and wide corolla tubes, which are suitable for bee pollination, but the long and narrow tubes of *P*. *axillaris* flowers are associated with hawkmoth pollination. At the metabolite level, the color (anthocyanin and other metabolites) and scent (benzenoids and phenylpropanoids) differ between these species and are implicated in pollinator attraction. Several studies of volatile composition and biosynthesis of *Petunia* species have been performed [3–5]. For example, isoeugenol synthase (IGS) and eugenol synthase (EGS) were identified and characterized. Mutation of the gene encoding IGS affect the spectrum of volatiles produced [3]. During flower development, such morphological and metabolic differentiation require tight spatial and temporal regulation at the cellular level via changes in cell division, cellular metabolism, and others. In most cases, the orchestrated differentiation is regulated by plant hormones. During metabolic differentiation, various metabolites, including biosynthetic intermediates and end-products, move from cell to cell or organelle to organelle. However, we know little about the molecular mechanisms underlying these differentiations, especially for transport of metabolites.

Most metabolites are transported across the membrane via membrane transporters. Recent analyses have identified various transporter families [6–11]. For example, carbohydrates are transported between different organelles or cells and are translocated from organs that act as energy sources to those that are sinks. SUT/SUC (sucrose transporters or sucrose carriers) and SWEET (Sugars Will Eventually be Exported Transporters) are major transporters for sucrose [6]. SUT/SUC has 12 transmembrane domains (TMDs) and functions as a sucrose/H^+^ symporter. Therefore, the plasma membrane-type SUT/SUC uptakes sucrose from the apoplast [12]. By contrast, SWEET typically has seven TMDs and facilitates diffusion of sugars across cell membranes. SWEETs function in nectar secretion, plant-microbe interaction, and embryo development by sugar transport [6, 13, 14]. For other carbohydrates, some transporters have been identified, and NSTs (nucleotide sugar transporters) function for intracellular transport of nucleotide sugars into organelles. One representative is the URGT (UDP-Rha (rhamnose)/UDP-Gal (galactose) transporter) family. The *Arabidopsis* URGT1 ~6 are localized in the Golgi and transports UDP-Rha or UDP-Gal into the Golgi lumen [15]. Since transported sugars are used for biosynthesis of cell wall polysaccharides [16], this transport process is essential for plant growth and development.

Plant hormones also are transported among cells. Some transporter families, including the ABC (ATP-binding cassette), MATE (multidrug and toxic compound extrusion), NPF (Nitrate transporter 1/Peptide transporter family), PIN (PIN-FORMED proteins), and AUX/LAX (AUXIN1-RESISTANT1 (AUX1)/LIKE AUX1), are involved in hormone transport [9]. ABC proteins comprise a large protein family consisting of ABCA-ABCI (ABCH is absent in plants) subfamilies [7]. Most family members have 12 TMDs and two nucleotide-binding domains and function as transporters to a diverse set of substrates, including plant hormones, secondary metabolites, and lipidic monomers. Therefore, these proteins are implicated as playing an important role in plant development, defense mechanisms, and morphology, among others. NPF proteins also have 12 TMDs, and most members function as H^+^/substrate co-transporters [10]. Their substrates include nitrate, plant hormones, such as auxin, ABA and gibellerin, and secondary metabolites, like glucosinolates. Therefore, members of the NPF family are involved in nitrate homeostasis, hormone transport, and translocation of secondary metabolites [10]. PIN and AUX/LAX function as auxin efflux and influx proteins, respectively [17].

Despite these progresses, a comprehensive understanding of morphological differentiation and metabolite transport during flower development and volatile production is still lacking. In *Petunia* species, previous studies reported transcriptome changes of *P*. *hybrida* flowers in response to pollination or ethylene [18, 19], however, little is known about differentially expressed genes (DEGs) for flower development from leaves to flowers, and transport proteins responsible for hormones and other substrates in *P*. *axillaris*. In this paper, we performed RNA-seq (RNA-sequencing) from flower cells of *P*. *axillaris* subsp. *parodii*, which emits a mixture of several benzenoids and phenylpropenes, and some enzymatic genes were characterized [3, 20]. Analysis of DEGs with gene ontology (GO) and Kyoto Encyclopedia of Genes and Genomes (KEGG) revealed genes enriched in the corolla limb for signaling pathways of hormones, such as gibberelin, as well as genes in the phenylpropanoid pathway for volatile and lignin formation. In addition, five transporters were identified with high expression in limbs, and their spatiotemporal expression patterns and physiological functions were analyzed and discussed. These results aid in providing a comprehensive characterization of the genes associated with morphological and metabolic differentiations in flower cells, and they generate new insight into the molecular mechanisms of transporter-dependent metabolite movement that is essential for organ development and secondary metabolite production.

## Results and discussion

### Transcriptome profiling of *P*. *axillaris* flowers

To investigate the genetics of morphological differentiation and metabolite transport during flower development of *P*. *axillaris* flower, we performed transcriptome analysis (RNA-seq) to compare transcript levels in corolla limbs to those of leaves and flower buds (stage2 of flower development). After *de novo* assembly, 53,243 unigene sequences were obtained with an average length of 1,199 bp and N50 of 1,572 bp. The obtained unigene sequences were annotated based on a homology search performed using BLASTX [21]. In total, 21,769 and 26,449 unigenes were annotated based on comparison to the TAIR10 and refseq databases, respectively (S1 Table).

### GO and KEGG analysis of DEGs in limbs

**Fig 1:**
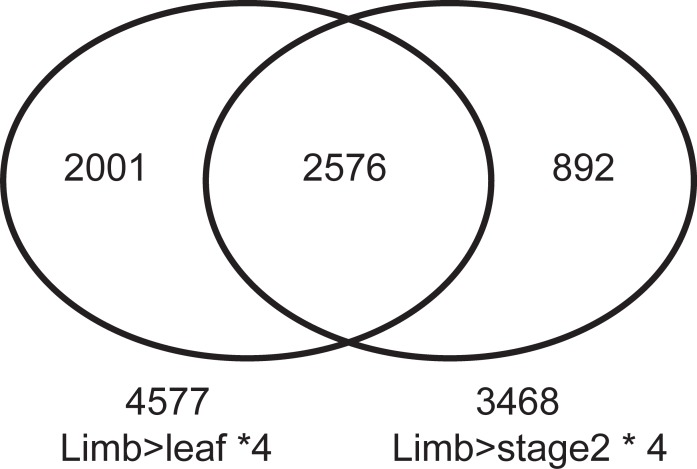
Venn diagram showing the number of up-regulated genes in limbs (greater than four-fold higher expression than in leaves or buds (stage2), and RPKM ≥ 1).

**Fig 2:**
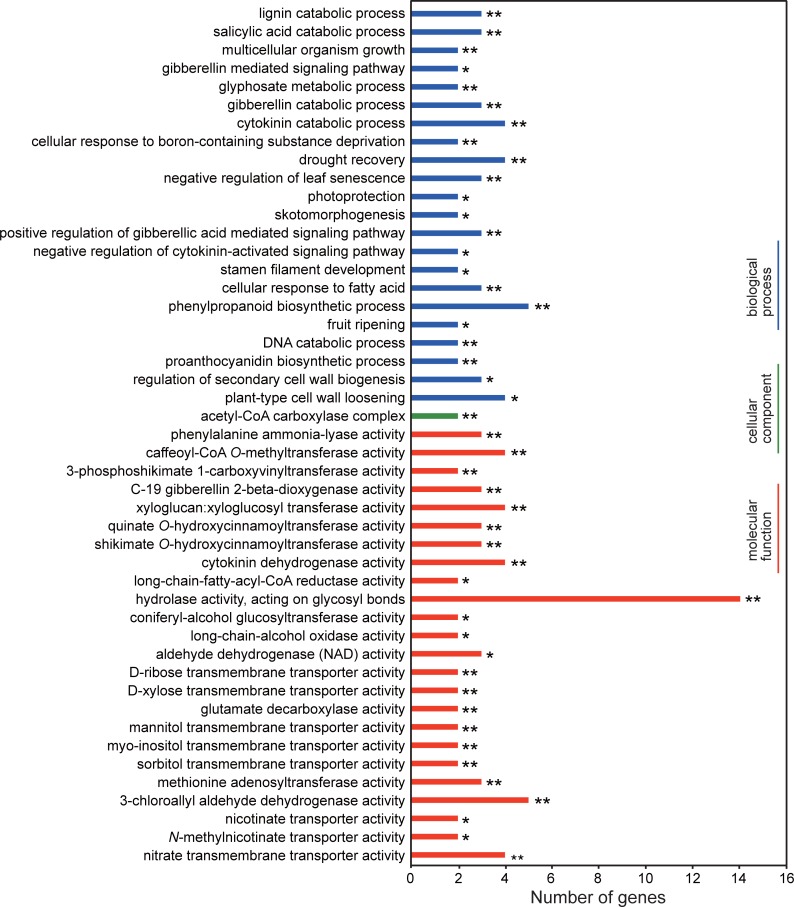
GO categories of DEGs in limbs compared with leaves and buds (stage2). Results are summarized under three main GO categories: biological process, cellular component, and molecular function. Fisher’s exact test was used for statistical analysis (*P < 0.05; **P < 0.01).

To identify DEGs in limbs, we compared RPKM values of unigenes among leaves, buds (stage2), and limbs, and found that 2,576 unigenes were expressed highly in limbs (four-fold or greater change; Fig 1). These unigenes were analyzed further using GO and KEGG to investigate the processes and pathways enriched in limbs (Fig 2 and Table 1). GO enrichment analysis (Fig 2) showed that 22 subcategories were enriched in the ‘‘Biological process” category, including the phenylpropanoid biosynthetic process (GO:0009699), drought recovery (GO:0009819), plant-type cell wall loosening (GO:0009828), cytokinin catabolic process (GO:0009823), lignin catabolic process (GO:0046274), salicylic acid catabolic process (GO:0046244), gibberellin catabolic process (GO:0045487), negative regulation of leaf senescence (GO:1900056), positive regulation of gibberellic acid mediated signaling pathway (GO:0009939), cellular response to fatty acid (GO:0071398), and regulation of secondary cell wall biogenesis (GO:2000652). In the ‘‘Cellular component”, acetyl-CoA carboxylase complex (GO:0009317) was enriched. In the ‘‘Molecular function”, 24 subcategories were enriched, including hydrolase activity, acting on glycosyl bonds (GO:0016798), 3-chloroallyl aldehyde dehydrogenase activity (GO:0004028), caffeoyl-CoA *O*-methyltransferase activity (GO:0042409), xyloglucan:xyloglucosyl transferase activity (GO:0016762), cytokinin dehydrogenase activity (GO:0019139), and nitrate transmembrane transporter activity (GO:0015112). In addition, we also identified biological pathways enriched in limbs by KEGG analysis (Table 1). There were 11 subcategories enriched, including phenylpropanoid biosynthesis (ath00940), phenylalanine metabolism (ath00360), phenylalanine, tyrosine and tryptophan biosynthesis (ath00400), stilbenoid, diarylheptanoid and gingerol biosynthesis (ath00945), flavonoid biosynthesis (ath00941), valine, leucine and isoleucine degradation (ath00280), and fatty acid degradation (ath00071). These data suggest that regulation and catabolism of hormones, such as cytokinin and gibberellin, play important roles in floral differentiation. Moreover, many metabolic processes, including phenylpropanoid biosynthesis, lignin biosynthesis and fatty acid metabolism, were highly enriched.

**Table 1:** Significantly enriched KEGG pathway of DEGs in limbs of *P*. *axillaris*.

Pathway	Annotation	No. of genes	*P*-value
Phenylpropanoid biosynthesis	ath00940	28	**
Phenylalanine metabolism	ath00360	14	**
Phenylalanine, tyrosine and tryptophan biosynthesis	ath00400	12	**
Stilbenoid, diarylheptanoid and gingerol biosynthesis	ath00945	12	**
Flavonoid biosynthesis	ath00941	11	**
Valine, leucine and isoleucine degradation	ath00280	10	**
Fatty acid degradation	ath00071	10	**
Butanoate metabolism	ath00650	6	**
Limonene and pinene degradation	ath00903	6	**
Carotenoid biosynthesis	ath00906	6	*
Zeatin biosynthesis	ath00908	4	*

Fisher’s exact test was used for statistical analysis (*P < 0.05; **P < 0.01).

We also found that 3,949 unigenes were down-regulated in limbs (1/4-fold or less change; S1 Fig). GO and KEGG pathway analyses showed that genes related to photosynthesis were decreased in limbs (S2 Table). These results suggest that buds (stage2) express many genes for photosynthesis and those genes decreased during flowering.

### Identification of genes involved in volatile biosynthesis

**Fig 3:**
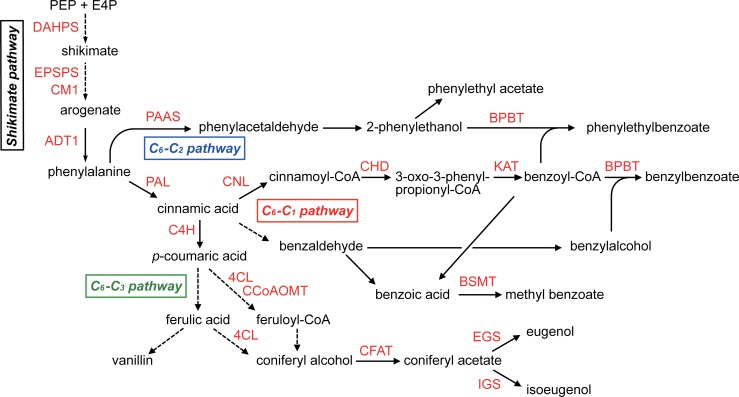
Putative VBP biosynthesis pathway in *P*. *axillalis*. VBPs are biosynthesized via the shikimate pathway, and C_6_-C_1_, C_6_-C_2_ and C_6_-C_3_ pathways. Biosynthetic enzymes, e.g., 3-deoxy-D-arabino-heptulosonate-7-phosphate synthase (DAHPS), 5-enolpyruvylshikimate-3-phosphate synthase (EPSPS), chorismate mutase 1 (CM1), arogenate dehydratase (ADT), phenylacetaldehyde synthase (PAAS), phenylalanine ammonia lyase (PAL), cinnamate 4-hydroxylase (C4H), 4-coumarate: CoA ligase (4CL), caffeoyl-CoA *O*-methyltransferase (CCoAOMT), coniferyl alcohol acetyltransferase (CFAT), isoeugenol synthase (IGS), eugenol synthase (EGS), cinnamoyl-CoA ligase (CNL), cinnamoyl-CoA hydratase/dehydrogenase (CHD), 3-ketoacyl-CoA thiolase (KAT), benzoyl-CoA:benzyl alcohol/2-phenylethanol benzoyltransferase (BPBT), *S*-adenosyl-L-methionine:benzoic acid/salicylic acid carboxyl methyltransferase (BSMT), are involved. Black continuous lines represent one enzymatic step, whereas black dotted lines represent multiple enzymatic steps.

**Fig 4:**
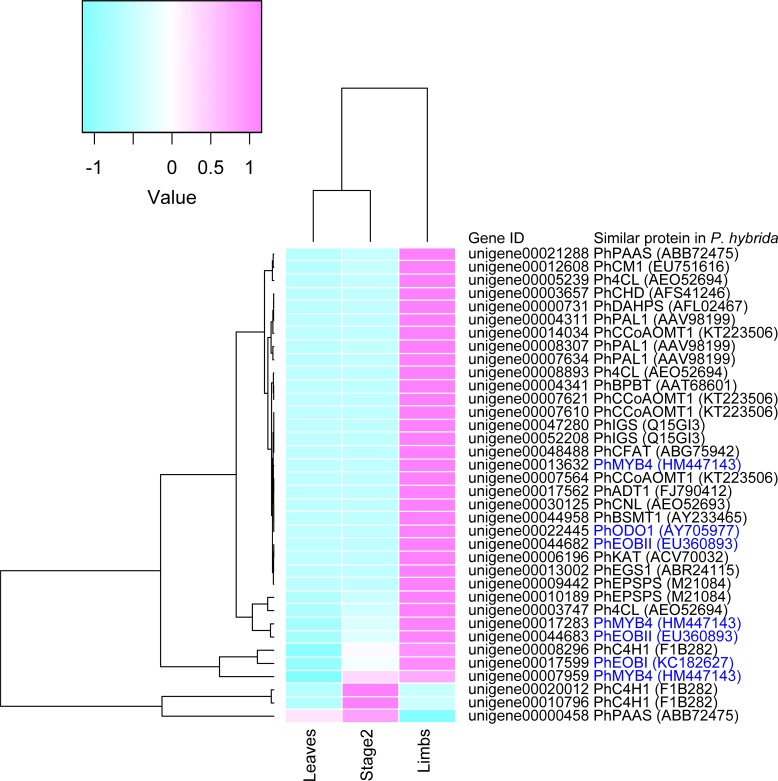
Expression profile of the candidate unigenes for VBP transcription factors and biosynthetic enzymes. Red indicates up-regulation and blue indicates down-regulation of unigenes. Transcription factors are shown in blue characters.

Most volatiles of *Petunia* species are synthesized via the phenylpropanoid pathway and are known as VBPs (volatile benzenoid/phenylpropanoids). The enzymes and transcription factors responsible for the biosynthesis of VBPs have been studied and characterized from other *Petunia* species, especially *P*. *hybrida* [22–24]. The predicted pathway is shown in Fig 3. Briefly, phenylalanine is converted via enzymes in the shikimate pathway (including DHAPS, EPSPS, CM1, and ADT1), and several enzymes, including PAL, produce various phenylpropanoids, such as isoeugenol, eugenol, and benzyl benzoate. Expression of some genes is regulated by transcription factors such as EOBI, EOBII, ODO1, and MYB [5, 24]. From the RNA-seq data of *P*. *axillalis* subsp. *parodii*, we successfully identified 37 unigenes encoding these proteins. The expression profiles of these are shown as a heat map (Fig 4). Most of these genes showed higher expression in limbs compared with buds and leaves, which is consistent with previous reports of biosynthetic genes in *P*. *hybrida* [22–24]. Therefore, most genes also are likely involved in VBP biosynthesis in the flowers of *P*. *axillalis* subsp. *parodii*.

### Identification and spatiotemporal expression analysis of transporter genes involved in morphological differentiation and metabolite transport

**Fig 5:**
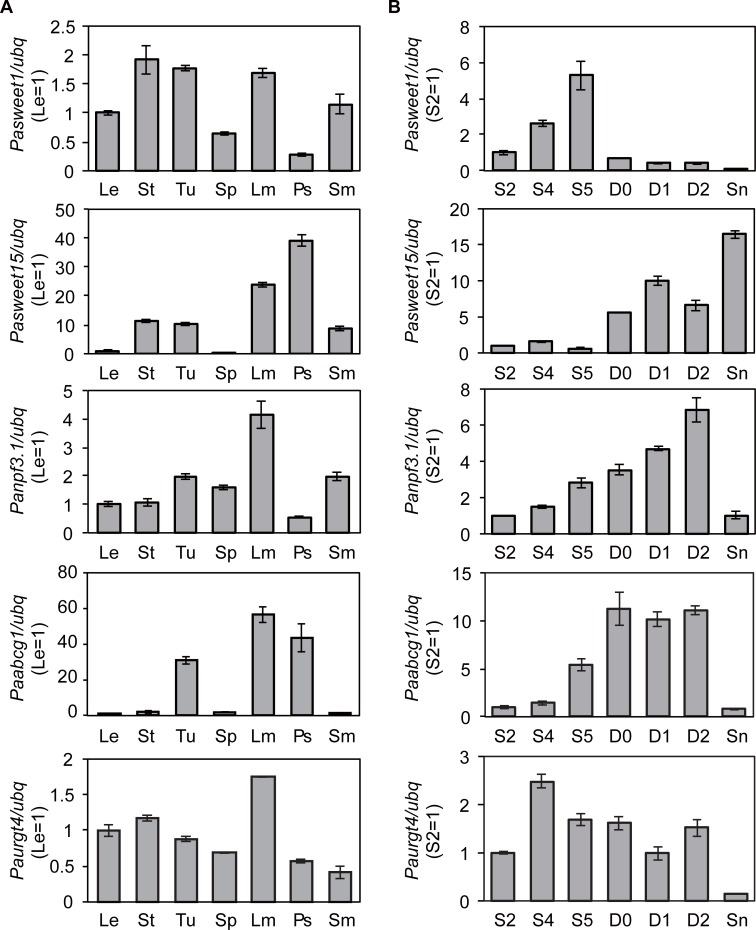
Spatiotemporal expression profiles of transporter genes by quantitative real-time RT-PCR. Expression analysis in different organs (A) and during flower development (B). Le, leaves; St, stems; Tu, tubes; Sp, sepals; Lm, limbs; Ps, pistils; Sm, stamens; S2, stage2; S4, stage4; S5, stage5; D0, day0; D1, day1; D2, day2; Sn, senescence. Relative expression levels are shown as fold change values. Results are mean ± SD of triplicates. The reference gene *ubq* was used to normalize the expression values. Similar expression profile was observed when *ran1* was used as another reference gene (S3 Fig).

To identify the transporters involved in morphological differentiation and metabolite transport in flowers, we first identified transporters expressed at high levels in limbs from RNA-seq data and then analyzed those expression profiles using quantitative real-time reverse transcription PCR. From RPKM analysis, five transporter genes were found to be expressed at high levels in limbs compared with leaves and buds (S3 Table). Two genes (Petunia01_unigene00003171 and Petunia01_unigene00031991) belonged to SWEET, whereas the others belonged to the NPF family (Petunia01_unigene00005960), G-type ABC transporter family (Petunia01_unigene00028220), and the URGT family (Petunia01_unigene00019148). By combining the genome sequence from the web database with genomic PCR and Sanger sequencing, we isolated their genomic sequences and full-length cDNAs (S2 Fig). PCR primers specific for each gene were prepared and quantitative RT-PCR was performed from RNA extracted from several organs (leaves, stems, tubes, sepals, limbs, pistils, and stamens) and flower developmental stages (stage2, stage4, stage5, day0, day1, day2, and senescence) (Fig 5 and S3 Fig).

The full-length cDNA of unigene00003171 was 1,570 bp and encoded a putative polypeptide consisting of 250 amino acids. The full-length cDNA of unigene00031991 was 1,587 bp, encoding a predicted protein of 321 amino acids. The prediction program for transmembrane regions suggested both proteins contain seven TMDs, which is consistent with it being a member of the SWEET family (S4 Fig). Plasma membrane or ER localization was predicted for two proteins (PSORT prediction program; http://psort1.hgc.jp/form.html). The amino acid sequences of unigene00003171 and 00031991 showed relatively high amino acid identity with AtSWEET1 (At1g21460) (67%) and AtSWEET15 (At5g13170) (46%) (S5 Fig). Therefore, we designated Petunia01_unigene00003171 as *Pasweet1* and Petunia01_unigene00031991 as *Pasweet15*. These proteins showed low similarity to PhNEC1, a *Petunia* SWEET protein involved in nectar secretion [13, 25]. *Pasweet1* was expressed at high levels in stems, tubes, and limbs, whereas it showed low expression in pistils (Fig 5A). Its mRNA expression increased gradually throughout bud development but decreased after flower opening (Fig 5B). Since AtSWEET1 functions as a plasma membrane-localized glucose transporter [26], PaSWEET1 might be involved in supplying glucose or other carbohydrates to tissues with high expression. In limbs, transported carbohydrates can be used as energy for flower opening and volatile biosynthesis. *Pasweet15* was expressed 10-fold higher in stems, tubes, and stamens compared with leaves, 30-fold higher than in limbs, and 40-fold higher than in pistils (Fig 5A). Its expression was low prior to flower opening and increased gradually after anthesis before reaching its maximum at the senescence (Fig 5B). AtSWEET15, AtSWEET11, and AtSWEET12 are expressed at high levels in embryos and are essential for seed development by providing sucrose to the embryo from the seed coat and endosperm [14]. Preferential expression in pistils and senescence suggested that PaSWEET15 also has a similar function to AtSWEET15.

The full-length cDNA of Petunia01_unigene00005960 was 2,080 bp, encoding a protein with 578 amino acids. Its putative structure has 12 TMDs (S4 Fig) and is predicted to localize to the plasma membrane. A putative translated product showed high amino acid identity with AtNPF3.1 (At1g68570) (73%) (S6 Fig), and we designated the full-length cDNA of unigene00005960 as *Panpf3*.*1*. Its mRNA was expressed at high levels in limbs, and slightly higher in tubes, sepals, and stamens (Fig 5A). This gene expression increased gradually until day2 but decreased significantly at senescence (Fig 5B). AtNPF3.1 imports gibberellin into the cytosol at the plasma membrane [27, 28]. Gibberellin regulates flower opening in plants [29], and volatile production in *Petunia* [30]. GO terms involved in gibberellin response and catabolism were highly enriched in limbs (Fig 2 and Table 1), which indicates that gibberellin signaling plays an important role in *P*. *axillaris*. These results suggest that PaNPF3.1 is involved in flower opening and volatile production in *Petunia* by transporting gibberellin into the cytosol from the apoplast.

The fourth identified transporter gene was unigene00028220, the full-length cDNA of which was 2,840 bp encoding a predicted polypeptide with 732 amino acids. This protein has one nucleotide-binding domain at its N-terminus and six TMDs at its C-terminus (S4 Fig). This represents a typical structure of a half-size G-type ABC transporter, and we designated this gene product as PaABCG1. This gene showed tissue-specific expression, with high expression only in tubes, limbs, and pistils (Fig 5A). Its expression increased gradually toward anthesis, maintained high expression during anthesis, and then decreased at senescence (Fig 5B). Although recent findings showed that PhABCG1 from *P*. *hybrida* facilitated volatile emission from flowers [31], most half-size ABCG proteins are involved in the formation of cutin, wax, and suberin [11]. PaABCG1 showed high amino acid identity with NtWBC1 (83%) from *Nicotiana tabacum* [32], StABCG1 (77%) from potato [33], AtABCG1, 2, 6, 16, and 20 (65–71%) from *A*. *thaliania* [34], and OsABCG5 from rice (60%) [35] (S7 Fig). Almost all of these, except for NtWBC1, are involved in suberin formation. Despite 23% amino acid identity to AtABCG13, the expression profile of PaABCG1 (limbs and pistils) is similar to AtABCG13 (petal and carpel), an essential protein for cuticle secretion in petals [36]. In addition, genes for fatty acids and drought recovery related to cuticle and suberin formation were up-regulated in limbs (Fig 2). These findings indicate that PaABCG1 might be involved in the formation of the cuticle or suberin, which functions in apoplastic diffusion barriers against drought stress.

The fifth transporter gene is the full-length cDNA of unigene00019148 (1,328 bp), which encodes a putative polypeptide 338 amino acids long. Its predicted structure has eight TMDs (S4 Fig). This gene product was designated as PaURGT4 because of its high amino acid identity with AtURGT4 (At4g39390) (77%), AtURGT6 (At1g34020) (74%), and AtURGT5 (At4g09810) (73%) and phylogenetic relationship between *Arabidopsis* URGT and related NSTs (S8 Fig). This gene was expressed ubiquitously in the tissues tested and showed slightly higher expression in limbs (Fig 5A). Across developmental stages, its expression was enhanced two-fold at the stage4, maintained during anthesis, before decreasing significantly (Fig 5B). AtURGT4 ~6 transport UDP-Gal or UDP-Rha into the lumen of the Golgi apparatus and ER as an exchanger to UMP (uridine monophosphate) [15]. Galactose is an important component of cell walls. Polysaccharides, synthesized from galactose and other carbohydrates in the Golgi lumen, are delivered from the Golgi to the apoplast via exocytosis [16]. During flowering, cell division and cell wall biosynthesis are active, as suggested from our GO and KEGG analysis (Fig 2 and Table 1), and galactose accumulates in the cell walls of *Petunia* flower [37]. Therefore, PaURGT4 likely is involved in cell wall biosynthesis by supplying galactose to the Golgi lumen, leading to morphological differentiation in flowers.

## Conclusions

Transcriptome analysis from *P*. *axillaris* limbs, buds, and leaves identified 53,243 unigenes, with 2,576 unigenes highly expressed in limbs. GO and KEGG analyses of DEG clarified that genes involved in hormone response, drought tolerance, and phenylpropanoid biosynthesis were enriched and implicated in morphological and metabolic differentiation in the flower. Several candidate genes for VBP transcription factors and biosynthetic enzymes were identified, which would be useful for characterization of VBP biosynthesis in *P*. *axillaris*. In addition, five transporter genes highly expressed in limbs were identified. Spatiotemporal expression analysis revealed their distinct expression profiles, with a possible involvement of these transporters in morphological differentiation and metabolite transport. PaNPF3.1 might be involved in flower opening and volatile production through its gibberellin transport. PaSWEET1 would supply sugars as energy for flowering and volatile biosynthesis. PaSWEET15 might play an important role in volatile production and seed production through its sucrose transport. PaABCG1 would function in the formation of apoplastic diffusion barrier like cutin and suberin by transport of lipidic compounds. PaURGT4 would transport galactose into Golgi lumen, which leads to cell wall biosynthesis. These findings provide a foundation to further the comprehensive identification of the genes involved in organ development and metabolite production, and they further elucidate the dynamics of metabolite transport in plant cells producing secondary metabolites.

## Materials and methods

### Plant materials

Seeds of *Petunia axillaris* subsp. *parodii* (kindly provided by Dr. Eran Pichersky, University of Michigan) were sown on soil (Tanemakibaido; Takii Seed Co., Ltd., Kyoto, Japan) and grown for 3 months in a culture room under 14 h of light and 10 h of dark at 25°C. The leaves, stems, tubes, sepals, limbs, pistils, and stamens were collected from three independent plants (at two months old). Flower stages (stage2, stage4, stage5, day0, day1, day2, and senescence) were defined previously [38] and sampled. Harvested samples were used to perform gene expression analysis.

### RNA-seq analysis

RNA-seq was performed as described previously [39] with minor modifications. Briefly, total RNA was extracted from limbs, bud (stage2), and leaves of *P*. *axillaris* using an RNeasy Plant Mini Kit (Qiagen, USA). RNA quality was evaluated using the BioAnalyzer 2100 (Agilent Technologies, USA). A 10 μg aliquot of total RNA was used to construct a cDNA library using the Illumina TruSeq Prep Kit v2, according to the manufacturer’s protocol (Illumina, USA). The resulting cDNA library was sequenced using the HiSeq 1500 (Illumina) in high output mode with 100 bp paired-end reads. Reads were assembled using the CLC Genomics Workbench version 5.5.2 (CLC Bio, Japan) with the following parameters: minimum contig length of 400 bp, and scaffolding after adaptor sequences and low quality reads were removed. For each sample, the reads were aligned to obtain Reads Per Kilobase of exon per Million mapped reads (RPKM). All raw read sequences are available on the NCBI sequence read archive under accession number, DRA006635.

### Annotation, DEG, GO, KEGG analysis

Obtained unigene sequences were annotated based on results of a homology search performed using BLASTX [40] against the *Arabidopsis thaliana* TAIR10 (https://www.arabidopsis.org/) and NCBI refseq protein databases, with an e-value cutoff of 1e-15. Unigenes satisfying the following: RPKM of Limb ≥ 1; RPKM of Limb > 4 x RPKM of Stage2; RPKM of Limb > 4 x RPKM of Leaf, were considered to be up-regulated unigenes. Unigenes satisfying the following: RPKM of Stage2 ≥ 1; RPKM of Limb < 1/4 x RPKM of Stage2; RPKM of Leaf ≥ 1; RPKM of Limb < 1/4 x RPKM of Leaf, were considered to be down-regulated unigenes. These DEGs were classified based on their GO (http://www.geneontology.org/) or KEGG metabolic pathway (http://www.genome.jp/kegg/) of the most similar proteins from *Arabidopsis thaliana* (e-value < 1e-15). The GO and pathway enrichment analyses were performed using Fisher’s exact test [41] relative to the entire set of unigenes. Hierarchical clustering analysis and heatmap generation was performed using R, version 3.4.4 [42].

### Cloning and sequence analysis of transporter genes with high expression in limbs

We searched for unigenes annotated as transporters from among the 500 top RPKM unigenes in limbs. From those unigenes, we also selected five unigenes that displayed RPKM values in limbs greater than those in leaves and stage2. For unigenes 00005960 and 00019148, we performed Rapid amplification of cDNA ends using a GeneRacer kit (Invitrogen, USA) to obtain the missing 5’ or 3’ cDNA sequence. PCR was performed using high-fidelity KOD-plus Neo DNA polymerase (TOYOBO, Japan). PCR products were cloned into pT7Blue T-vector (Novagen, USA) and their sequences were confirmed. The full-length cDNA sequences were deposited to the DDBJ database (Japan). The transmembrane regions of each transporter were predicted using the web program (TMHMM Server v. 2.0; http://www.cbs.dtu.dk/services/TMHMM/).

To obtain the genomic structure of each unigene, genomic DNA was extracted from a frozen stem using the BioMasher II (Nippi, Japan). Primers were designed to amplify each gene, PCR was performed using KOD-plus Neo DNA polymerase (TOYOBO, Japan), and their sequences were analyzed. Genome sequences were confirmed against the reference sequence of the *P*. *axillaris* genome [43] and Sol Genomics Network (https://solgenomics.net/organism/Petunia_axillaris/genome).

### RNA isolation and real-time quantitative reverse transcription PCR

Total RNA was prepared from seven tissues (i.e., leaves, stems, tubes, sepals, limbs, pistils, stamens), and seven developmental stages of flowers. Reverse transcription was conducted using the ReverTra Ace qPCR RT Master Mix with gDNA Remover (TOYOBO, Japan), according to manufacturer’s instructions. Primers were designed to amplify each gene separately. UBQ (Accession no. SGN-U207515) and RAN1 (Accession no. SGN-U207968) were used as an endogenous control, according to Mallona et al. [44]. Data were normalized against UBQ or RAN1. Primer sequences and PCR conditions are listed in S4 Table. Real-time PCR and data acquisition were performed using the LightCycler Nano instrument (Roche, Switzerland) and TOYOBO KOD SYBR qPCR Mix (TOYOBO, Japan).

## Supporting information

S1 Fig Venn diagram showing the number of down-regulated genes in limbs (less than 1/4-fold compared to leaves or buds (stage2), and RPKM of leaves or buds ≥ 1). (EPS)

S2 Fig Genomic structure of transporter genes and primer pairs for quantitative real-time RT-PCR. Introns are in black, exons are in pink, and UTRs are in blue.
(TIFF)

S3 Fig Spatiotemporal expression profiles of transporter genes by quantitative real-time RT-PCR. Expression analysis in different organs (A) and during flower development (B). Le, leaves; St, stems; Tu, tubes; Sp, sepals; Lm, limbs; Ps, pistils; Sm, stamens; S2, stage2; S4, stage4; S5, stage5; D0, day0; D1, day1; D2, day2; Sn, senescence. Relative expression levels are shown as fold change values. Results are mean ± SD of triplicates. The reference gene *ran1* was used to normalize the expression values.
(EPS)

S4 Fig Putative transmembrane domains of transporters. The membrane-spanning domains of transporters were predicted using the TMHMM program (http://www.cbs.dtu.dk/services/TMHMM/).
(EPS)

S5 Fig Phylogenetic relationship of SWEET families. PaSWEET1 (LC375808), PaSWEET15 (LC375809), AtSWEET1 (At1g21460), AtSWEET2 (At3g14770), AtSWEET3 (At5g53190), AtSWEET4 (At3g28007), AtSWEET5 (At5g62850), AtSWEET6 (At1g66770), AtSWEET7 (At4g10850), AtSWEET8 (At5g40260), AtSWEET9 (At2g39060), AtSWEET10 (At5g50790), AtSWEET11 (At3g48740), AtSWEET12 (At5g23660), AtSWEET13 (At5g50800), AtSWEET14 (At4g25010), AtSWEET15 (At5g13170), AtSWEET16 (At3g16690), AtSWEET17 (At4g15920).
Phylogenetic analyses were conducted in MEGA7 software (Kumar S., Stecher G., and Tamura K., Molecular Biology and Evolution 33:1870–1874 (2016)) using maximum likelihood and 1000 bootstrap resampling events. Bootstrap values (maximum 100) are shown at branching points. The scale bar shows the number of amino acid substitutions per site.
(EPS)

S6 Fig Phylogenetic relationship of NPF families. PaNPF3.1 (LC375810), AtNPF1.2 (At1g52190), AtNPF2.7 (At3g45650.1), AtNPF2.9 (At1g18880.1), AtNPF2.10 (At3g47960.1), AtNPF2.11 (At5g62680.1), AtNPF2.12 (At1g27080.1), AtNPF2.13 (At1g69870.1), AtNPF3.1 (At1g68570.1), AtNPF4.1 (At3g25260.1), AtNPF4.2 (At3g25280.1), AtNPF4.3 (At1g59740.1), AtNPF4.4 (At1g33440.1), AtNPF4.5 (At1g27040.1), AtNPF4.6 (At1g69850.1), AtNPF5.2 (At5g46050.1), AtNPF5.13 (At1g72125.1), AtNPF5.14 (At1g72120.1), AtNPF6.2 (At2g26690.1), AtNPF6.3 (At1g12110.1), AtNPF6.4 (At3g21670.1), AtNPF7.2 (At4g21680.1), AtNPF7.3 (At1g32450.1), AtNPF8.1 (At3g54140.1), AtNPF8.2 (At5g01180.1), AtNPF8.3 (At2g02040.1), AtNPF8.4 (At2g02020.1), AtNPF8.5 (At1g62200.1), OsNPF2.2 (Os12g44100.1), OsNPF4.1 (Os11g12740.1), OsNPF5.5 (Os10g33210.1), OsNPF7.1 (Os07g41250.1), OsNPF7.3 (Os04g50950.1), OsNPF7.4 (Os04g50940.1), OsNPF8.1 (Os01g04950.1), OsNPF8.2 (Os07g01070.1), OsNPF8.5 (Os03g51050.1), OsNPF8.9 (Os03g13274.2), OsNPF8.20 (Os06g49250.1), CsNPF3.2 (Z69370.2), MtNPF1.7 (Medtr1g009200.1), MtNPF6.8 (Medtr5g085850.1).
At: *A*. *thaliana*, Cs: *Cucumis sativus*, Mt: *Medicago truncatula*, Os: *Oryza sativa*, Pa: *Petunia axillaris*
Phylogenetic analyses were conducted in MEGA7 software (Kumar S., Stecher G., and Tamura K., Molecular Biology and Evolution 33:1870–1874 (2016)) using maximum likelihood and 1000 bootstrap resampling events. Bootstrap values (maximum 100) are shown at branching points. The scale bar shows the number of amino acid substitutions per site.
(EPS)

S7 Fig Phylogenetic relationship of ABCG families. PaABCG1 (LC375811), AtABCG1 (At2g39350), AtABCG2 (At2g37360), AtABCG3 (At2g28070), AtABCG4 (At4g25750), AtABCG5 (At2g13610), AtABCG6 (At5g13580), AtABCG7.1 (At2g01320.1), AtABCG7.2 (At2g01320.2), AtABCG7.3 (At2g01320.3), AtABCG7.4 (At2g01320.4), AtABCG8 (At5g52860), AtABCG9 (At4g27420), AtABCG10 (At1g53270), AtABCG11 (At1g17840), AtABCG12 (At1g51500), AtABCG13 (At1g51460), AtABCG14 (At1g31770), AtABCG15 (At3g21090), AtABCG16 (At3g55090), AtABCG17 (At3g55100), AtABCG18 (At3g55110), AtABCG19 (At3g55130), AtABCG20 (At3g53510), AtABCG21.1 (At3g25620.1), AtABCG21.2 (At3g25620.2), AtABCG22.1 (At5g06530.1), AtABCG22.2 (At5g06530.2), AtABCG22.3 (At5g06530.3), AtABCG23 (At5g19410), AtABCG24 (At1g53390), AtABCG25 (At1g71960), AtABCG26 (At3g13220), AtABCG27 (At3g52310), AtABCG28 (At5g60740), PpABCG7 (XP001775949.1), StABCG1 (XP006345915.1), NtWBC1 (AAR06252), SbWBC11 (Sb06g023280), PhABCG1 (JQ088099), OsABCG5 (Q8H8V7), OsABCG15 (Os06g40550), OsABCG26 (Os10g0494300), OsSTR1 (JN608807), OsSTR2 (JN608806).
At: *A*. *thaliana*, Nt: *Nicotiana tabacum*, Os: *Oryza sativa*, Pa: *Petunia axillaris*, Ph: *Petunia hybrida*, Pp: *Physcomitrella patens*, Sb: *Sorghum bicolor* St: *Solanum tuberosum*
Phylogenetic analyses were conducted in MEGA7 software (Kumar S., Stecher G., and Tamura K., Molecular Biology and Evolution 33:1870–1874 (2016)) using maximum likelihood and 1000 bootstrap resampling events. Bootstrap values (maximum 100) are shown at branching points. The scale bar shows the number of amino acid substitutions per site.
(EPS)

S8 Fig Phylogenetic relationship of URGT families. PaURGT4 (LC375812), AtURGT1 (At1g76670), AtURGT2 (At1g21070), AtURGT3 (At5g42420), AtURGT4 (At4g39390), AtURGT5 (At4g09810), AtURGT6 (At1g34020), AtUXT1 (At2g28315), AtUXT2 (At2g30460), AtUXT3 (At1g06890).
Phylogenetic analyses were conducted in MEGA7 software (Kumar S., Stecher G., and Tamura K., Molecular Biology and Evolution 33:1870–1874 (2016)) using maximum likelihood and 1000 bootstrap resampling events. Bootstrap values (maximum 100) are shown at branching points. The scale bar shows the number of amino acid substitutions per site.
(EPS)

S1 Table Summary of illumina transcriptome sequencing for leaves, stage2, and limbs of *P*. *axillalis*. (XLSX)

S2 Table GOs and KEGG pathway of significantly down-regulated genes in limbs of *P*. *axillaris*. (XLSX)

S3 Table List of transporter unigenes with high relative expression in limbs. (XLSX)

S4 Table Primer sets and PCR condition of cDNAs selected for quantitative RT-PCR. (XLSX)
