# Supplementary figures and images for: Transcriptome analysis of Petunia axillaris flowers reveals genes involved in morphological differentiation and metabolite transport
Source: PLoS One. 2018 Jun 14;13(6):e0198936. doi: 10.1371/journal.pone.0198936 (PMC6002047; doi:10.1371/journal.pone.0198936)

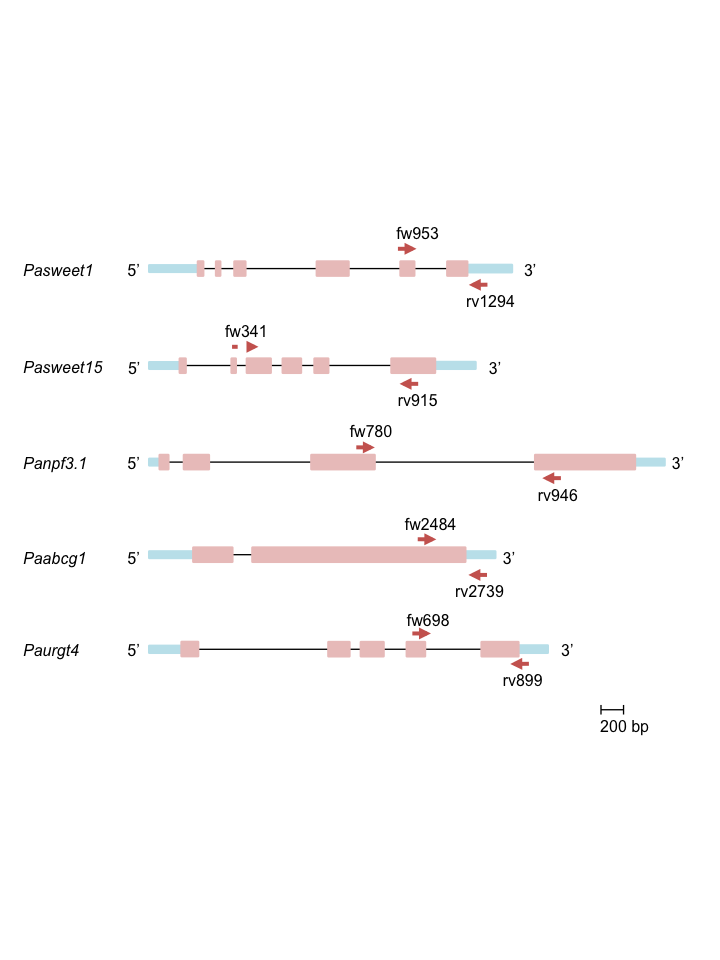

Supplement: S2 Fig — Introns are in black, exons are in pink, and UTRs are in blue. (TIFF) [file pone.0198936.s002.tiff]
